# Supplementary material for: 2D ferroelectric narrow-bandgap semiconductor Wurtzite’ type α-In2Se3 and its silicon-compatible growth
Source: Nat Commun. 2025 Aug 9;16:7364. doi: 10.1038/s41467-025-62822-7 (PMC12335484; doi:10.1038/s41467-025-62822-7)
Supplement: Supplementary file 1 — Supplementary Information [file 41467_2025_62822_MOESM1_ESM.pdf]

Supplementary Information for

2D ferroelectric narrow-bandgap semiconductor Wurtzite' type α-In_2Se_3 and its silicon-compatible growth

Yuxuan Jiang[1,2#], Xingkun Ning[3#], Renhui Liu[1,2#], Kepeng Song[4#], Sajjad Ali[5], Haoyue Deng[6], Yizhuo Li[1], Biaohong Huang[1], Jianhang Qiu[1], Xiaofei Zhu[1], Zhen Fan[6], Qiankun Li[7], Cheng-bing Qin[8,9], Fei Xue[10], Teng Yang[1,2]*, Bing Li[1,2], Gang Liu[1,2], Weijin Hu[1,2]*, Lain-Jong Li[11], Zhidong Zhang[1]

[1]Shenyang National Laboratory for Materials Science, Institute of Metal Research, Chinese Academy of Sciences, Shenyang 110016, China
[2]School of Materials Science and Engineering, University of Science and Technology of China, Shenyang 110016, China
[3]Hebei Key Lab of Optic-Electronic Information and Materials, National and Local Joint Engineering Research Center of Metrology Instrument and System, Hebei University, Baoding 071002, China
[4]Electron Microscopy Center, School of Chemistry & Chemistry Engineering, Shandong University, Jinan 250100, China.
[5]Energy, Water, and Environment Lab, College of Humanities and Sciences, Prince Sultan University, Riyadh 11586, Saudi Arabia.
[6]Institute for Advanced Materials and Guangdong Provincial Key Laboratory of Optical Information Materials and Technology, South China Academy of Advanced Optoelectronics, South China Normal University, Guangzhou 510006, China
[7]School of Physical Science and Technology, Jiangsu Key Laboratory of Frontier Material Physics and Devices, Soochow University, Suzhou 215006, China
[8]State Key Laboratory of Quantum Optics Technologies and Devices, Institute of Laser Spectroscopy, Shanxi University, Taiyuan 030006, China
[9]Collaborative Innovation Center of Extreme Optics, Shanxi University, Taiyuan 030006, China
[10]Center for Quantum Matter, School of Physics, Zhejiang University, Hangzhou 310058, China
[11]Department of Materials Science and Engineering, National University of Singapore, Singapore, Singapore.

#: These authors contribute equally to this work
*Email: yangteng@imr.ac.cn; wjhu@imr.ac.cn

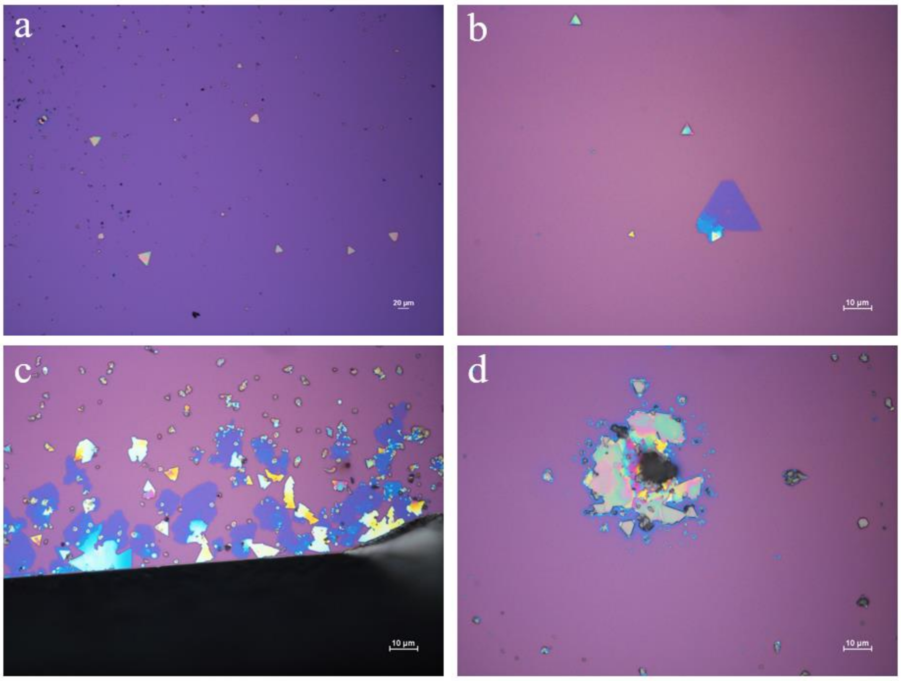

Figure S1. Optical images of In_2Se_3 nanosheets prepared by traditional CVD method. a Isolated In_2Se_3 nanosheets grown on a SiO_2 (300 nm)/Si substrate. The nanosheets are dispersed and cannot form large-area films. **b** The layer-plus-island growth mode of In_2Se_3. **c** In_2Se_3 nanosheets grown along the edge of the substrate, because the edge-step contact is thermodynamically favored[1]. **d** In_2Se_3 nanosheets grown around defects on the substrate.

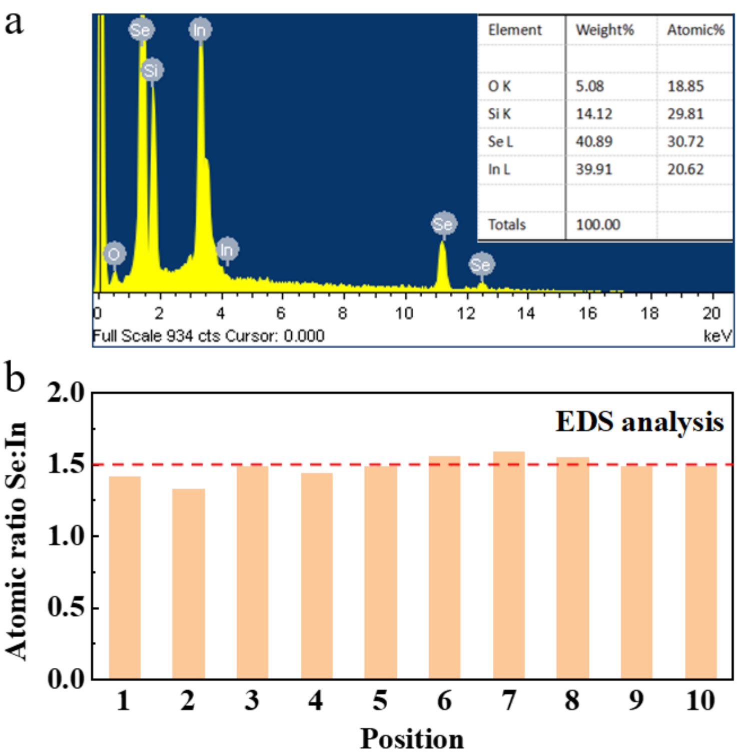

Figure S2. Elemental analysis of the WZ' type α-In_2Se_3 film. a Typical EDS curve. Inset

presents the corresponding element composition analysis from the EDS spectrum. **b** atomic ratio statistics of Se: In summarized from EDS curves acquired at 10 different positions on the film. The ratio of Sn: In is estimated to be 3:2 as expected for stoichiometric In_2Se_3.

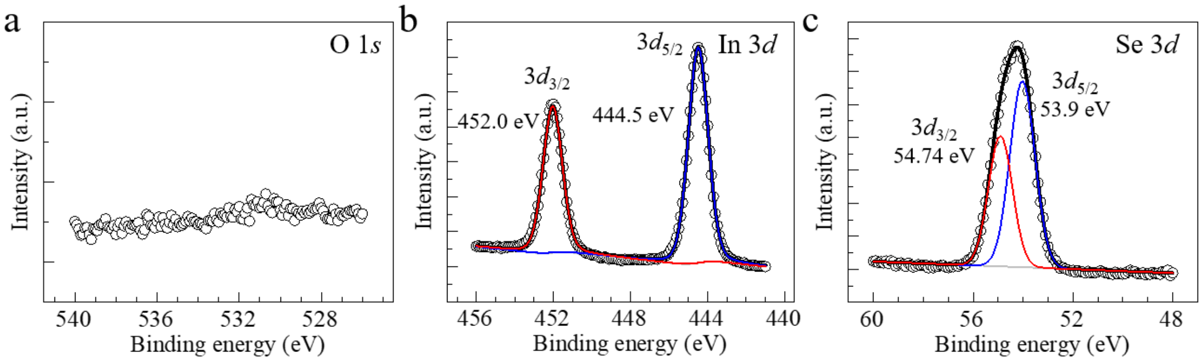

Figure S3. XPS spectra analysis of WZ' type *α*-In_2Se_3 film. a O 1*s*, **b** In 3*d*, and **c** Se 3*d* states. The absence of the characteristic peak of O1s indicates that In_2O_3 was completely converted to In_2Se_3 during the selenisation process. The binding energies of In $3d_{5/2}$ and In $3d_{3/2}$ are determined to be ~444.49 eV and ~452.04 eV, and binding energies of Se $3d_{5/2}$ and Se $3d_{3/2}$ are determined to be ~53.9 eV and ~54.74 eV. These values are close to that of *α*-In_2Se_3 with a ZB' structure[2].

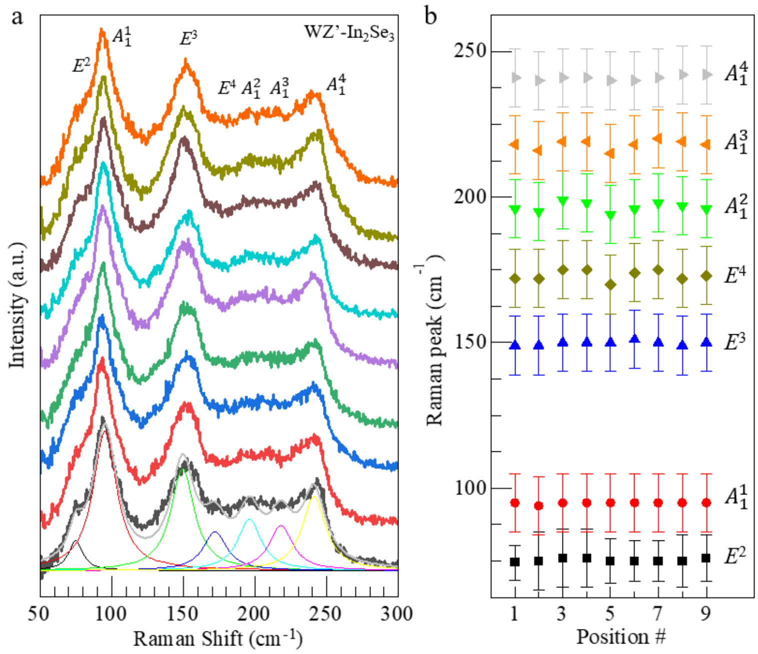

Figure S4. Raman spectra statistics. a. The Raman spectra acquired on 9 positions randomly selected in the film with the baseline being subtracted. The fitted peaks with Lorenz functions are shown at the bottom for a typical Raman spectrum at position #1. **b**. The statistics of the Raman peaks. The error bars represent the Full Width at Half Maximum (FWHM) of the Ramam peaks. Due to the overlapping, the peaks of E^4, A_1^2, and A_1^3 show more distinct fluctuations compared with other Raman peaks.

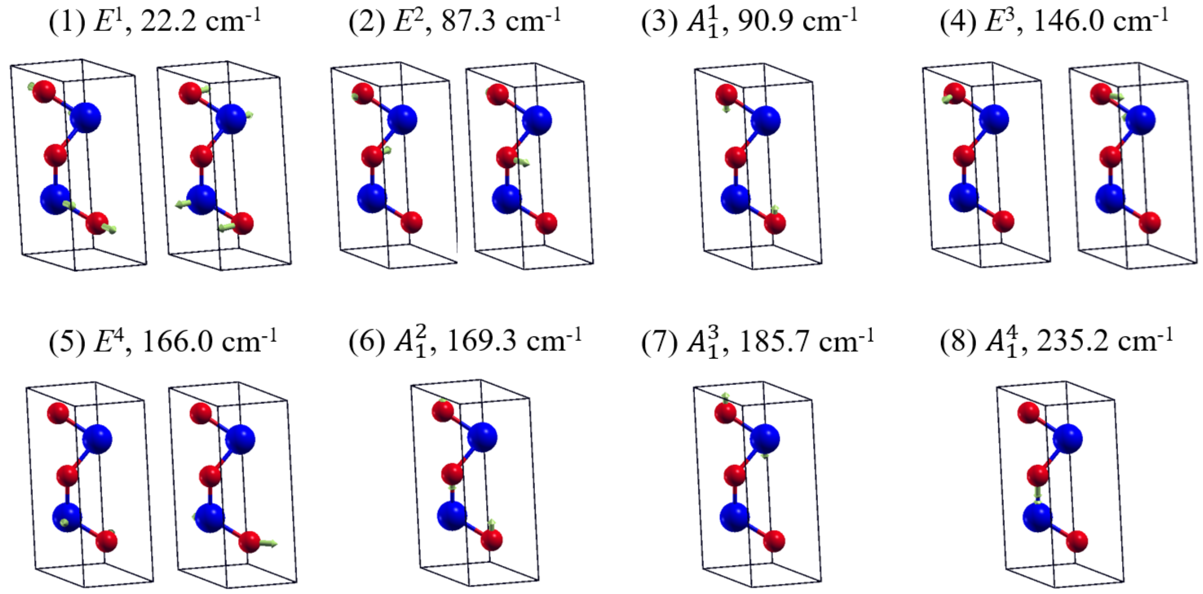

Figure S5. The vibration modes of ZB' type α-In_2Se_3 monolayer. The number, frequency and symmetry representation of each mode are marked above each subplot. The green arrows indicate the vibrational directions and magnitudes. (1) In-plane shearing mode of top and bottom In (Se) atoms; (2) In-plane shearing mode of top and middle Se atoms; (3) Interlayer breathing mode; (4) In-plane shearing mode of top Se and top In atoms; (5) In-plane shearing mode of bottom Se and bottom In atoms; (6) Stretching mode of all Se atoms; (7) Stretching mode of top, middle Se and top In atoms; (8) Stretching mode of middle, bottom Se and bottom In atoms.

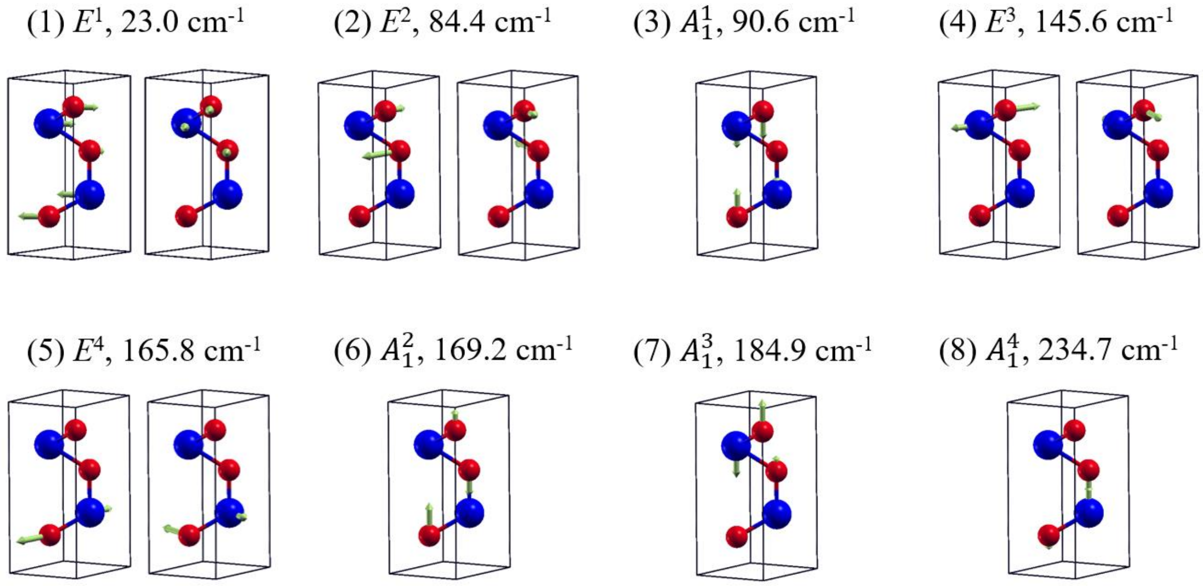

Figure S6. The vibration modes of WZ' type α-In_2Se_3 monolayer. The number, frequency and symmetry representation of each mode are marked above each subplot. The green arrows indicate the vibrational directions and magnitudes. (1) In-plane shearing mode of all atoms; (2) In-plane shearing mode of top and middle Se atoms; (3) Interlayer breathing mode; (4) In-plane shearing mode of top Se and top In atoms; (5) In-plane shearing mode of bottom Se and bottom

In atoms; (6) Stretching mode of all Se atoms; (7) Stretching mode of top, middle Se and top In atoms; (8) Stretching mode of middle, bottom Se and bottom In atoms.

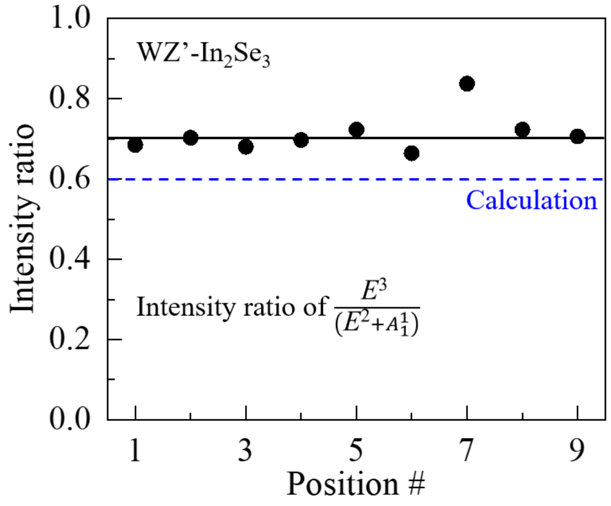

Figure S7. The intensity ratio of E^3 over that of E^2 and A_1^1 derived from experimental Raman spectra (solid circles) and theoretical spectra (blue dashed line).

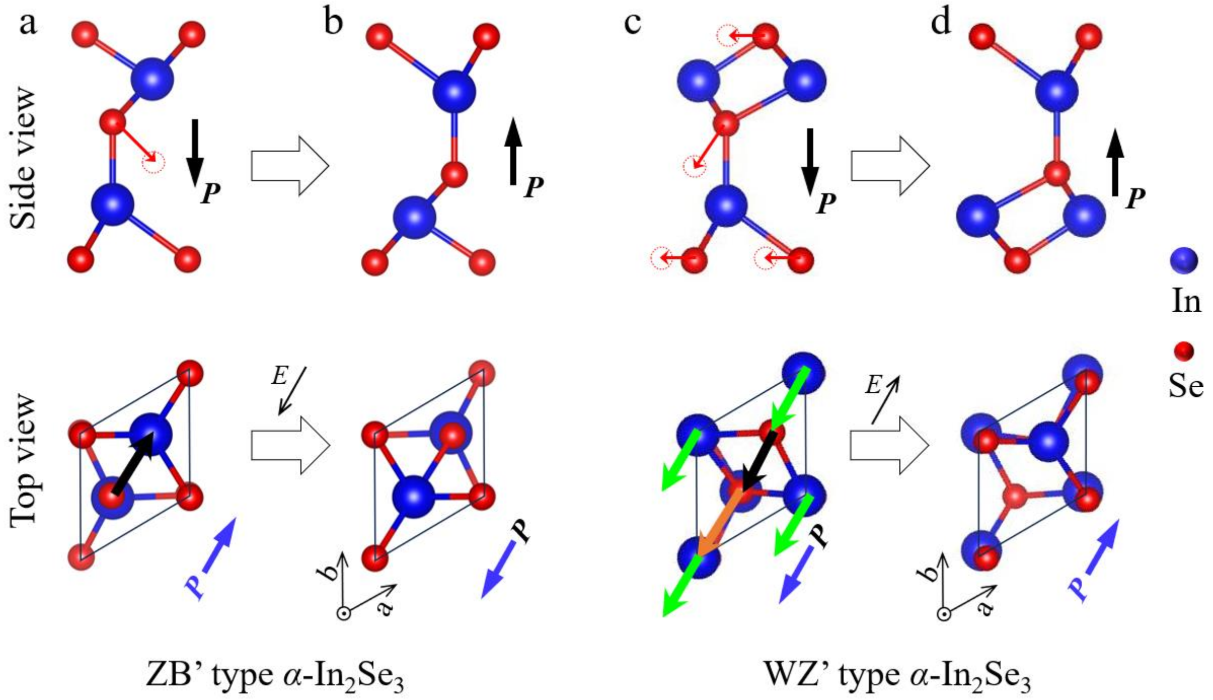

Figure S8. Structure and ferroelectricity of α-In_2Se_3. (a) ZB' phase with downward polarization, (b) ZB' phase with upward polarization, (c) WZ' phase with downward polarization, and (d) WZ' phase with upward polarization. Top panel, side view along [110]; bottom panel, top view along [001]. Arrows with P indicate the polarization, arrows denote the displacement direction of Se atoms, and dashed circles indicate the final position of Se atoms upon polarization reversal.

The switching of ferroelectric polarization in α-In_2Se_3 is achieved through the lateral and

vertical movement of Se atoms. In contrast to the minimal ion displacement of ~ 10 pm observed in conventional ferroelectric perovskites, the displacement in In_2Se_3 is significantly larger, reaching up to 100 pm, which is comparable to the lattice constants (~400 pm). Under these circumstances, the IP polarization can be analyzed within the context of fractional quantum ferroelectricity (FQFE), as proposed by J. Ji et al[3]. The concept of FQFE is based on an in-depth understanding of the modern theory of polarization, which expresses the polarization using a Berry phase approach. A unit charge e travels through the unit cell will cause polarization $\mathbf{Q} = \frac{e}{\Omega}\mathbf{R},$ with Ω the volume of the unit cell and **R** the lattice vector of the moving path. For ZB'-In_2Se_3 as shown in **Fig. S8a**, the movement of the central Se ions from position $(\frac{1}{3}\mathbf{a}, \frac{1}{3}\mathbf{b})$ to $(\frac{2}{3}\mathbf{a}, \frac{2}{3}\mathbf{b})$ accomplishes the IP polarization switching from [110] to [-1-10], where **a** and **b** are the lattice basis vectors. Therefore, $\mathbf{R} = \frac{1}{3}\mathbf{a} + \frac{1}{3}\mathbf{b},$ and $\Delta\mathbf{P} = \frac{2e}{3\Omega}(\mathbf{a} + \mathbf{b})$ taking into account that the Se ion carries two charges. By using a lattice constant of $a = b = 4.106$ Å and $c = 6.8$ Å[4], we derive $\Omega = 99.28$ Å^3 and $P = 38.2$ μC/cm^2. In the case of WZ' type α-In_2Se_3 as depicted in **Fig. S8c**, the concurrent movements of the top, central, and bottom Se ions are required to accomplish the polarization reversal with IP polarization switching from [-1-10] to [110]. Therefore, the IP polarization of the WZ' phase is three times greater than that of ZB' phase, reaching $P = 115$ μC/cm^2. It should be noted that the actual kinetic pathway for reversing the polarization might involve a more complex route, aimed at reducing the switching barrier. Nonetheless, the polarization value itself remains constant once the initial and final atomic configurations are established.

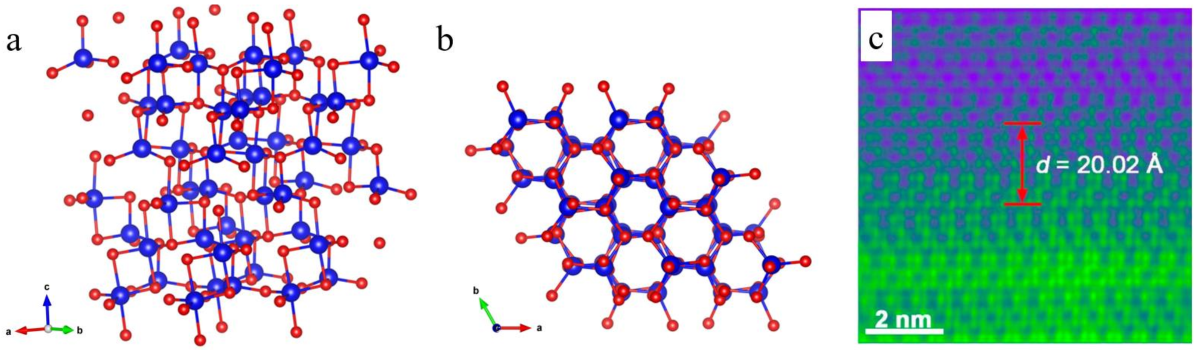

Figure S9. Crystal structures of γ-In_2Se_3. (a) Perspective view, (b) top view of γ-In_2Se_3. (c) HRTEM image along zone axis [010] [5].

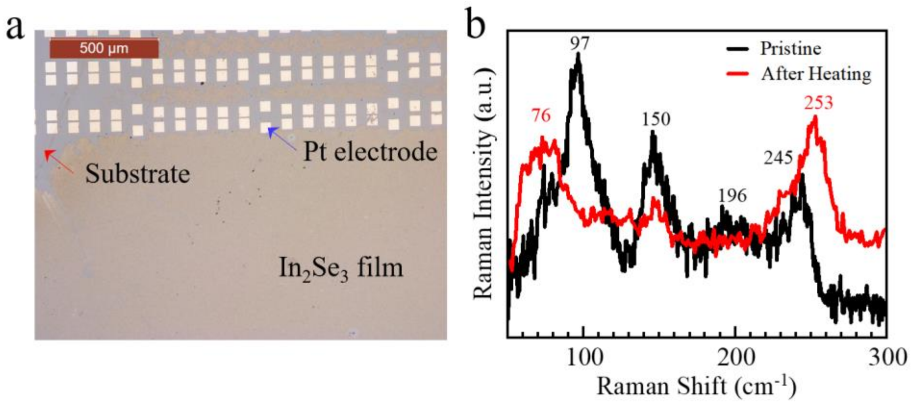

Figure S10. The optical image (a) and Raman spectra (b) of WZ' type α-In_2Se_3 film after SHG measurement. The optical image shows that some part of the sample, particularly the areas around Pt electrodes, evaporates after the warming and cooling process during the SHG measurement. Combined with the sharp decrease of the SHG signal at 620 K (**Fig. 2f**), this fact suggests that an annealing temperature of 620 K will damage the thin film sample. Pt as an active catalyst, may react with WZ' type α-In_2Se_3 at high temperatures, accelerating its decomposition or evaporation, which needs investigation in the future. The Raman spectra at room temperature after the SHG measurement (red) are distinctly different from that of the pristine film (black), characterized by the disappearance of the peaks at 97 cm^{-1} and 245 cm^{-1}. While the newly emerging peak at 253 cm^{-1} is usually attributed to glassy Se[6]. These results indicate that WZ'-α-In_2Se_3 film decomposes at high temperatures during the SHG measurement.

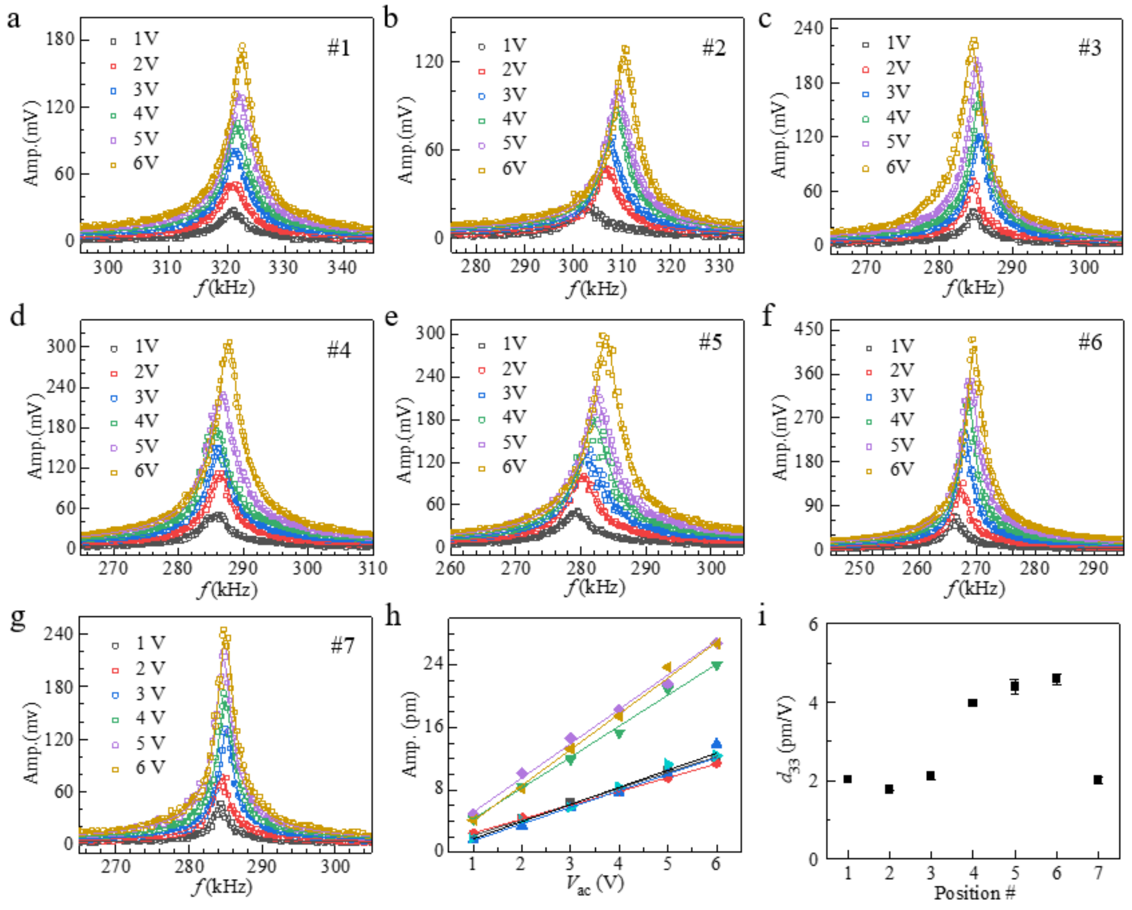

Figure S11. Piezoelectric coefficient d_{33} of WZ' type α-In_2Se_3 film. a-g. The piezoelectric response near the contact resonant frequency under different driving voltages on seven randomly selected positions in the film. Solid lines are fittings according to the harmonic oscillator model. **h.** The piezoelectric amplitude in unit of pm as a function of the driving voltage derived from **a-g**. **i**. d_{33} values derived from the slope of **h**. The error bars represent the standard error in linear fittings.

Strong piezoelectric resonant peaks have been observed for WZ' type α-In_2Se_3 film, with the resonant peak amplitude increasing with the external tip voltages. The shape of the peak can be described using the following harmonic oscillator model:[7]

$$A(\omega) = A_{\max}\omega_0^2/Q\sqrt{(\omega^2-\omega_0^2)^2+(\omega\omega_0/Q)^2}, \qquad (1)$$

where $\omega = 2\pi f$ is the angular frequency of the external voltage, $A_{\max}$ is the maximum resonant piezo-amplitude at resonant frequency ω_0, and Q is the quality factor of the probe-sample system. The experimental data can be well fitted by this equation (solid lines in **Figs. S11, a-g**), from which the piezoelectric coefficient d_{33} of the samples could be determined through the equation:

$$d_{33} = (A_{\max}/Q)/V_{ac}\,, \qquad (2)$$

where V_{ac} is the amplitude of the applied voltage onto the probe. **Fig. S11h** shows that $A_{\max}/Q$ is linearly proportional to V_{ac}, and the d_{33} values are derived to be 2.00 - 5 pm/V (**Fig. S11i**). This value is comparable with other 2D ferroelectrics including ZB'-In_2Se_3 (20nm, d_{33}~2.8 pm/V)[8], $CuInP_2S_6$ (0.72nm, d_{33}~5.12 pm/V)[9], 3R MoS_2 (18nm, d_{33}~0.88 pm/V)[10], and InSe (120 nm, d_{33}~4 pm/V)[11], etc.

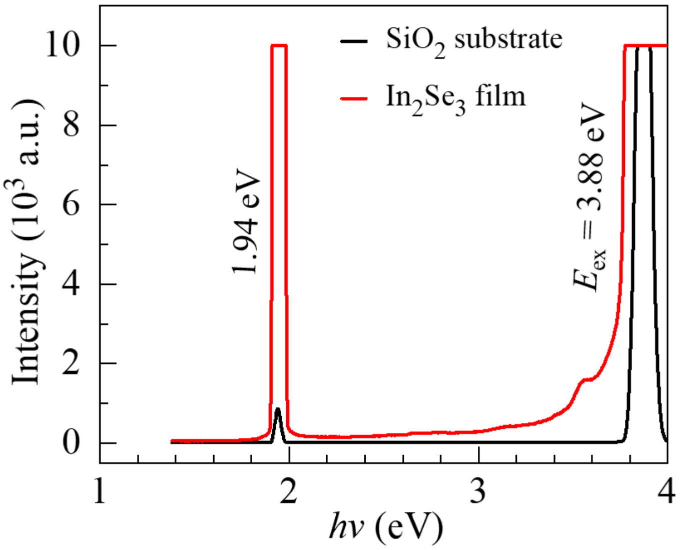

Figure S12. Photoluminescence (PL) spectra of WZ' type α-In_2Se_3 film. An excitation photon energy of 3.88 eV (wavelength of 320 nm) has been used. The peak located at 1.94 eV originates from the grating interference of the light source, which has also been observed for the SiO_2/Si substrate. No PL peak was detected within the photon energy range of 1.4 eV to 3.4 eV, indicating that the direct E_g of 3.1 eV derived from the empirical Tauc plot is not favored for WZ'-α-In_2Se_3.

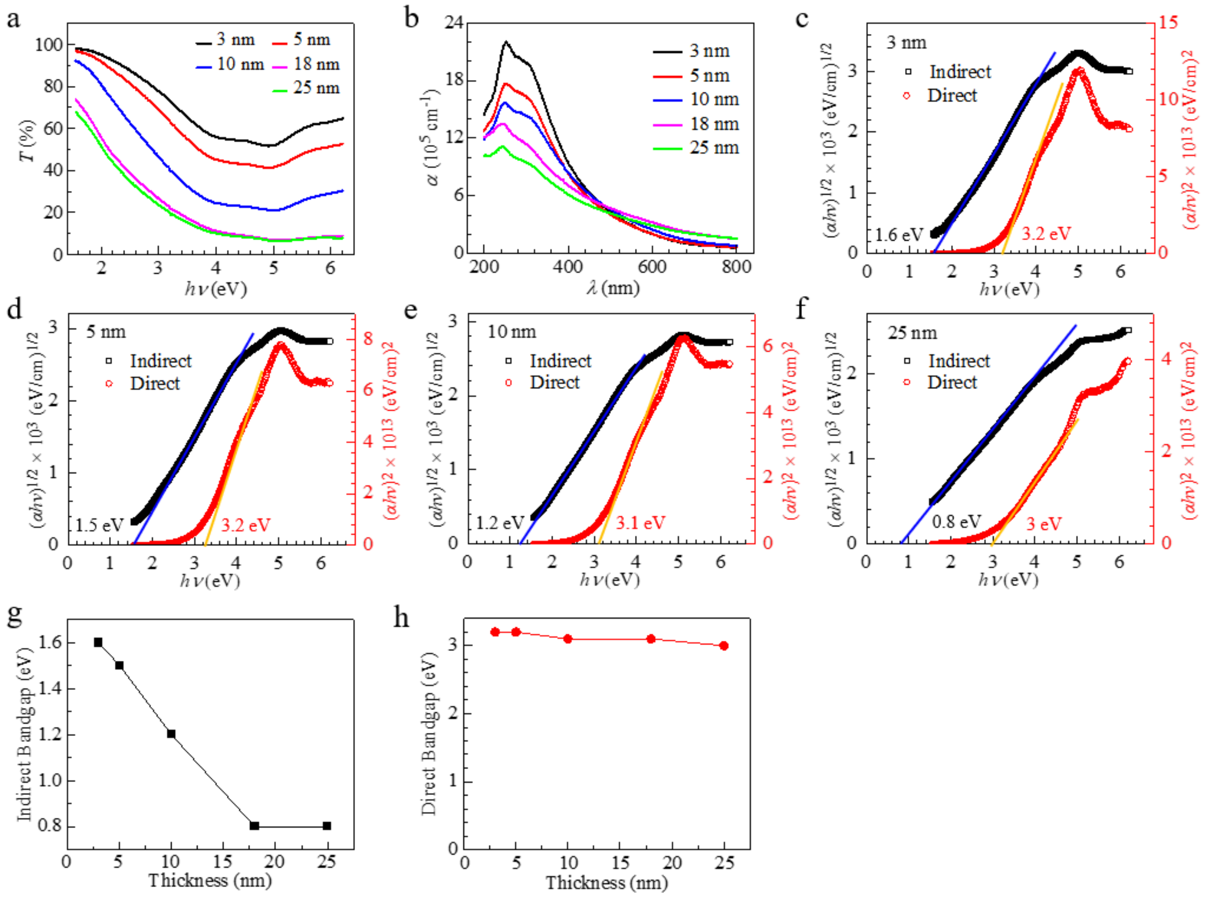

Figure S13. Thickness dependent optical properties of WZ'-type α-In$_2$Se$_3$. a The transmission spectrum within the wavelength range of 200 nm to 800 nm. **b** The as derived optical absorption coefficient as a function of wavelength. **c-f** $(\alpha h\nu)^{1/2}$ (left) and $(\alpha h\nu)^2$ (right) Tauc plots to determine the band gaps for films with thickness ranging from 3 nm to 25 nm. Solid lines are the fittings. **g** The as-derived indirect bandgaps, and **h** The direct bandgaps.

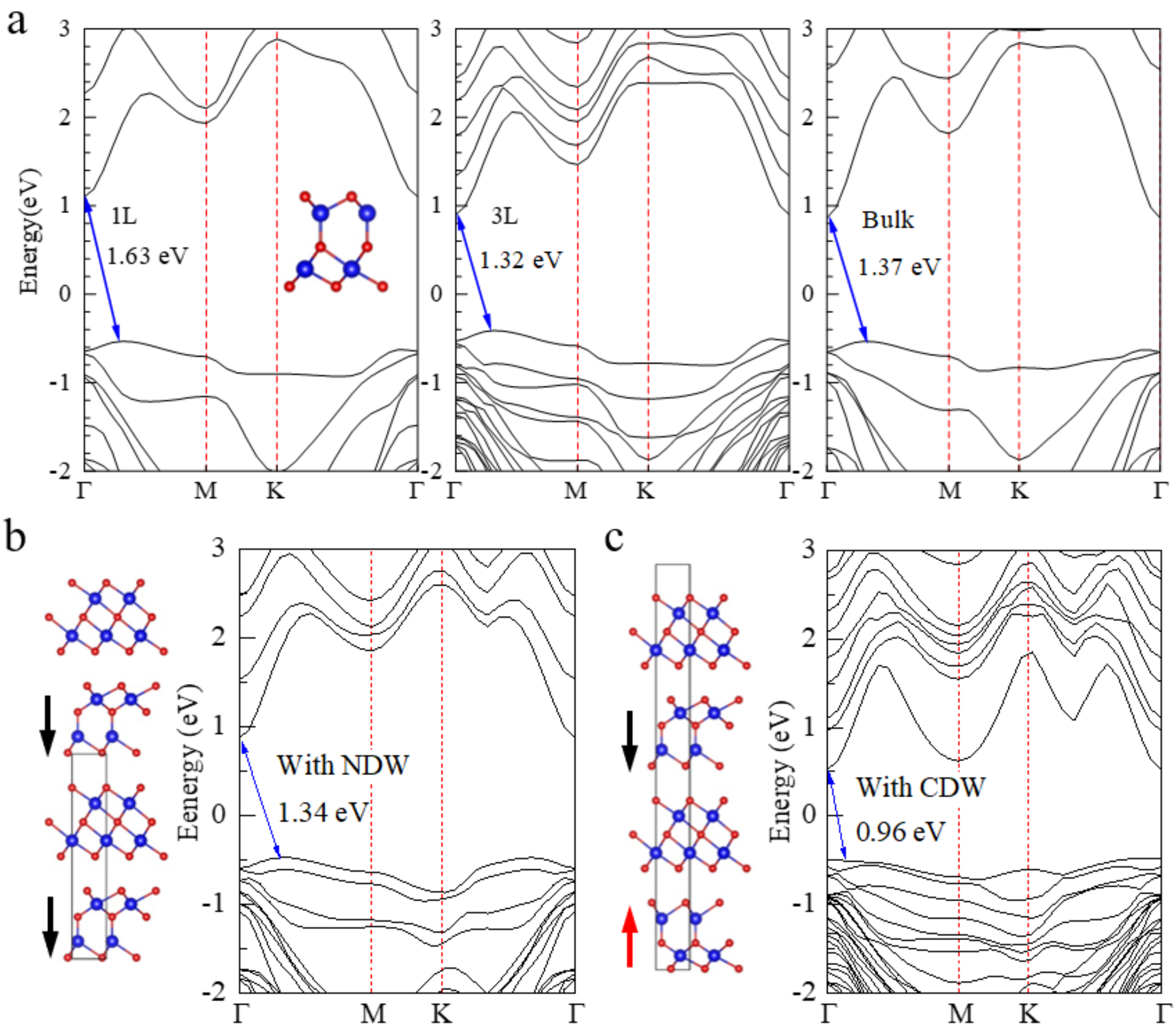

Figure S14. Calculated electronic band structures of WZ' type *α*-In_2Se_3. (a) 1L, 3L, and bulk without considering the domain wall. (b) after considering the neutral domain wall (NDW). NDW is sandwiched between two neighbouring WZ' layers with the same polarization direction. (c) after considering the charged domain wall (CDW). CDW is sandwiched between two neighbouring WZ' layers with opposite polarization directions.

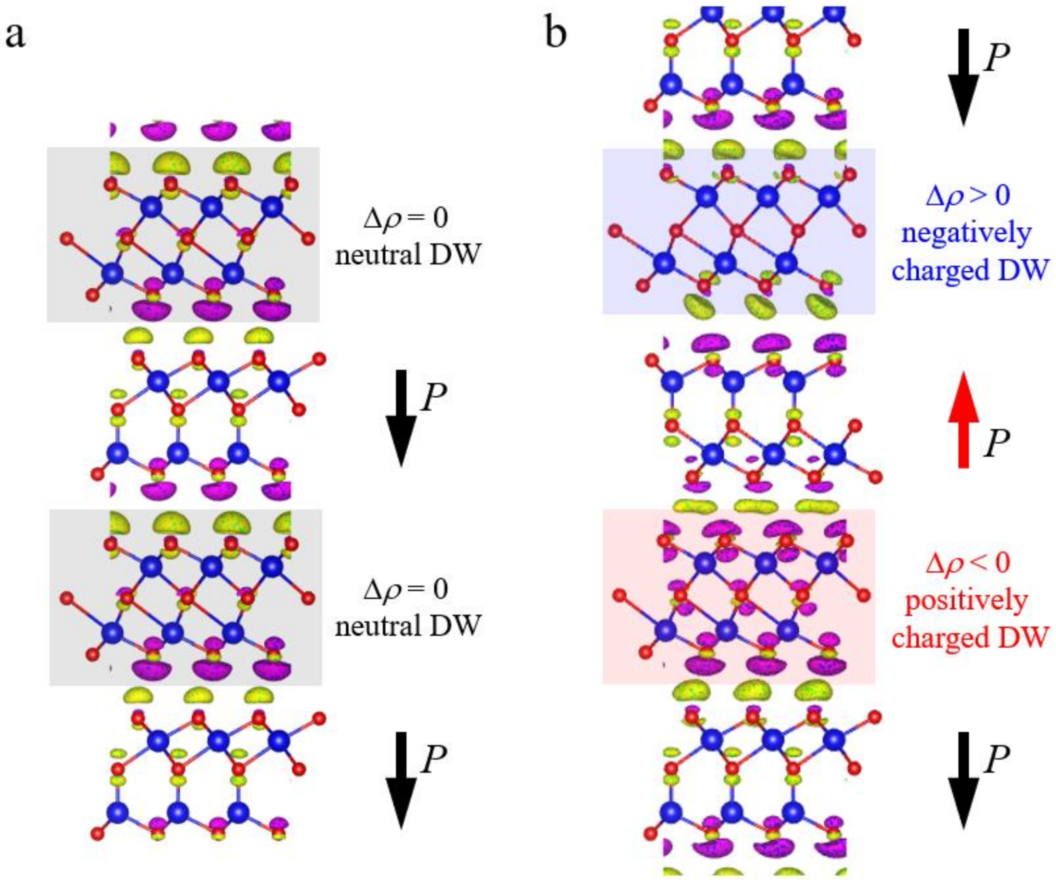

Figure S15. Calculated interlayer charge re-distribution Δ*ρ* of bulk WZ' type *α*-In_2Se_3 by considering (a) the neutral domain walls (NDWs), and (b) the charged domain walls (CDWs). $\Delta\rho = \rho$(WZ' + DW layers) - ρ(WZ' layer) - ρ(DW layer). The isosurface charge density in the figure is ~8.6×10^{-5} e/Bohr^3. The electrostatic columbic interaction between the two CDWs with opposite charges lower down the energy, and thereby stabilize the CDW system.

CDW is typically considered unstable due to its high depolarization field and associated electrostatic energy. In conventional ferroelectrics like $BiFeO_3$, CDW can be stabilized via the charge screening, structure reconstruction, or domain wall movement. In our case, the total energy of the WZ' phase with CDW (~ -3.632 eV /atom) is slightly higher than that of NDW (~ -3.634 eV/atom), suggesting instability. However, we find that electrostatic interactions between neighbouring CDWs with opposite charges can reduce the energy by ~ 4 meV/atom, thereby stabilizing the CDW state (**Fig. S15**). Specifically, this attractive Coulomb energy gain from NDW to CDW can be estimated by $Q^2/(4\pi\varepsilon_r)$, where CDW net charge $Q = \rho\times(4/3)\pi d^3$ with $\rho \sim 8.6\times10^{-5}$ e/Bohr^3, $d \sim 2.66$ Å (bond length of In-Se), the inter-CDW distance $r \sim 15$ Å. While this long-range inter-CDW energy gain may go beyond the Coulomb Ewald sum, it remains significant in terms of the energy precision of 10^{-5} eV in our calculation.

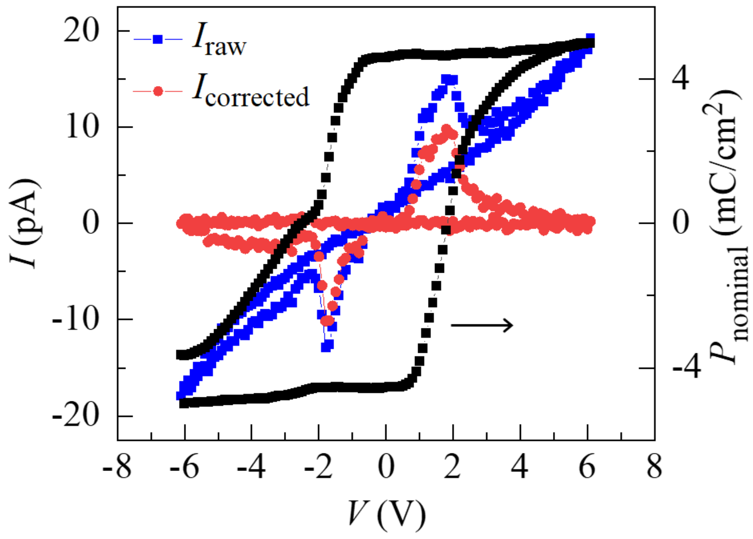

Figure S16. Nominal polarization ($P_{nominal}$) derived from the *I-V* curve. By extracting the leakage current whose magnitude increases linearly with the applied voltage from the raw current (I_{raw}, blue square), we obtained the switching current (I_{sw}, red circle) as a function of voltage. The $P_{nominal}$-V hysteresis loop (right) is then constructed by integration I_{sw} over time. We get a $P_{nominal}$ of around 5 mC/cm^2 which is far beyond the theoretically calculated in-plane ferroelectric polarization of WZ' type α-In_2Se_3 (P~115 μC/cm^2)[4], and typical 3D ferroelectrics such as $BaTiO_3$ (P~26 μC/cm^2)[12]. This suggests that the current peaks are not only from the displacement current of FE polarization switching but also associated with the accompanying charge injection and subsequent trapping at the Pt/In_2Se_3 interface as we discussed in the main text.

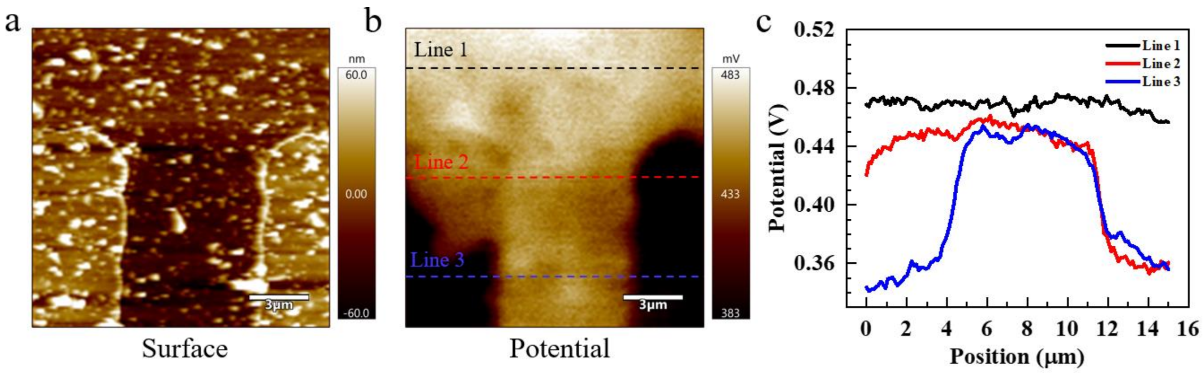

Figure S17. Scanning Kelvin Probe Force Microscopy (SKPFM) results analysis. a The surface image around a pair of Pt electrodes on the film. **b** The corresponding SKPFM image. **c** the specific potential distribution curves along the dashed lines in **b**. In the SKPFM amplitude image, the Pt electrode will display a relatively low potential compared to that of In_2Se_3 because of its high work function. However, an abnormal region on the electrode exhibits a potential increase of ~ 0.1 V, which can be ascribed to charge trapping at the In_2Se_3/Pt interface, as previously discussed in the text in relation to **Fig. 4d**.

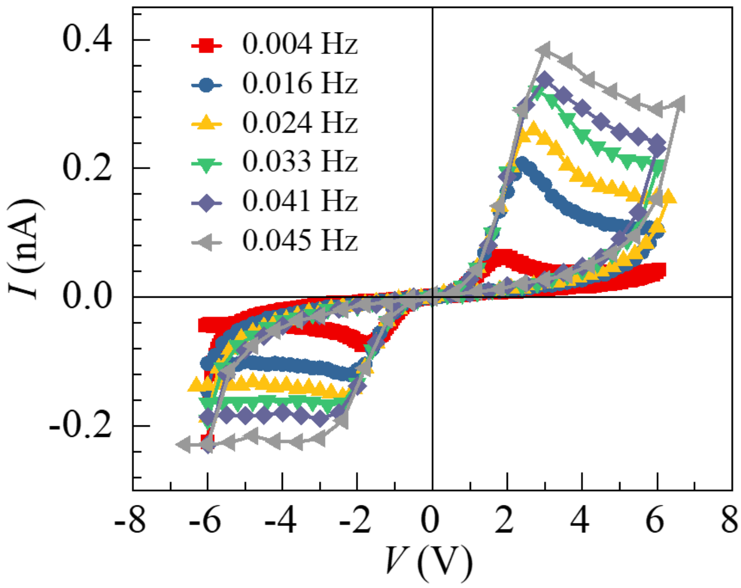

Figure S18. The durability of *I-V* curves for Pt/ WZ' type *α*-In_2Se_3/Pt IP device. Following 180 days of storage at room temperature within a dry cabinet, the *I-V* curves for the device continue to exhibit the current switching behaviour, which underscores the exceptional stability of the films.

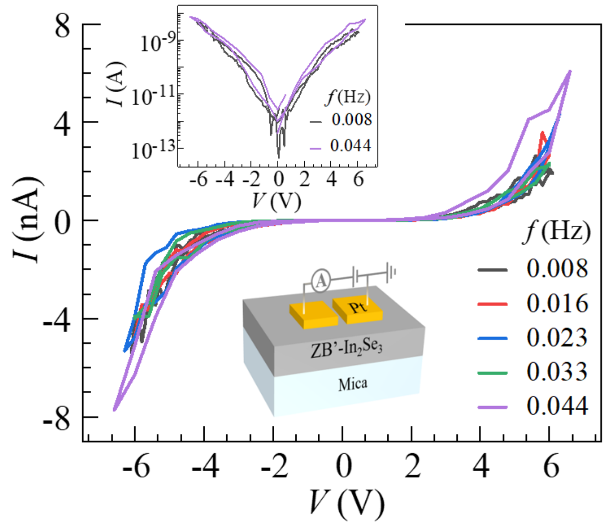

Figure S19. *I-V* characteristics of two terminal devices based on ZB' type *α*-In_2Se_3 films. Various frequencies ranging from 0.008 Hz to 0.044 Hz have been used. The up-inset shows the typical *I-V* curves in semi-log scale. The down-inset schematically shows the device structure and measurement configuration. Minor current switching peaks have been observed.

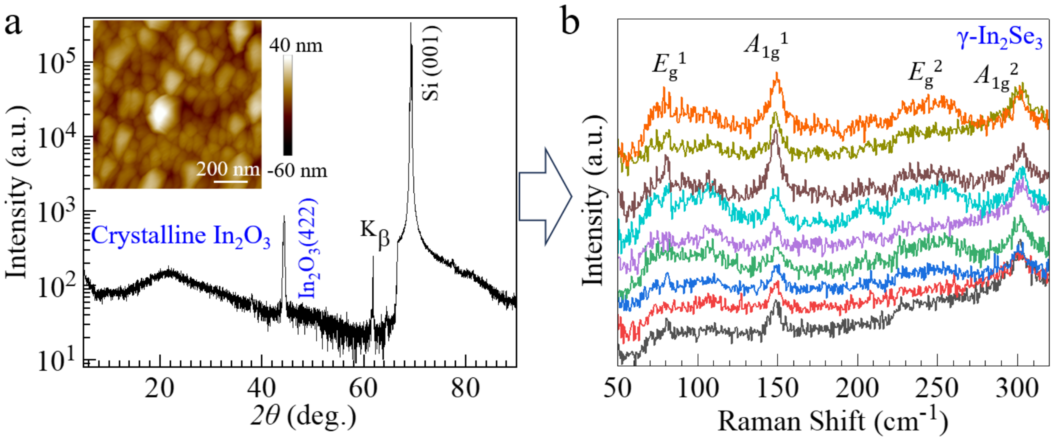

Figure S20. In-situ transport growth of In_2Se_3 by combining PLD and CVD and using crystalline In_2O_3 as the precursor. (a) The XRD of crystalline In_2O_3 prepared by PLD and (b) the Raman spectra of films after selenization. From the Raman peaks, we identify the film to be γ- In_2Se_3. This is different from the WZ'-type α-In_2Se_3 as presented in the manuscript when using amorphous In_2O_3 as the precursor.

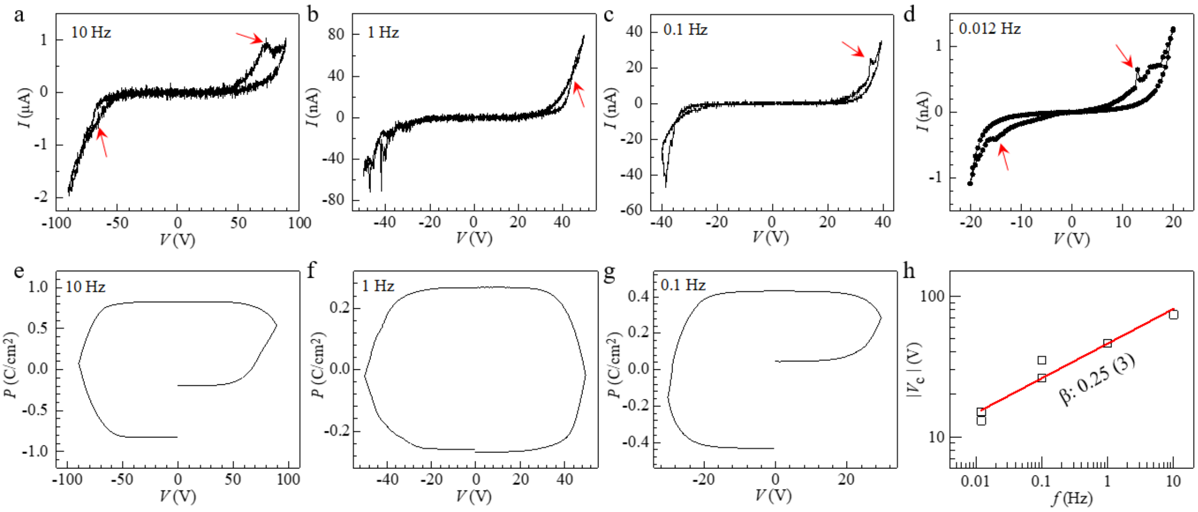

Figure S21. *I-V* curves and the corresponding *P-E* loops measured by ferroelectric tester. a-c. Transient current loops at 10 Hz, 1 Hz, and 0.1 Hz measured by a ferroelectric tester. d. Quasistatic *I-V* curve measured by Source Meter for comparison. e-g. The corresponding *P-E* loops at 10 Hz, 1 Hz, and 0.1 Hz. h. The coercive voltage (V_c) at different frequencies. V_c is determined from the kink of *I-V* curves. Red solid line is the fitting according to the power law $V_c \sim f^{\beta}$ with β equaling to 0.25(3).

Table S1. Crystal structure parameters for γ-In_2Se_3[13] and WZ' type α-In_2Se_3.

Phase	Dimension	Lattice constants	Refs.
γ-In_2Se_3	3D	$a = b = 7.123$ Å, $c = 19.39$ Å, $\gamma = 120°$	13
WZ' type α-In_2Se_3	2D	$a = b = 3.8$ Å, $c = 6.9$ Å, $\gamma = 120°$	This work

References

1 Andryushechkin, B. V., Cherkez, V. V., Pavlova, T. V., Zhidomirov, G. M. & Eltsov, K. N. Structural transformations of Cu(110) surface induced by adsorption of molecular chlorine. *Surf. Sci.* **608**, 135 (2013). https://doi.org/10.1016/j.susc.2012.10.005

2 Zhang, W., Su, Q., Zhang, B., Peng, J. & Li, Y. α-In_2Se_3 nanostructure-based photodetectors for tunable and broadband Response. *ACS Applied Nano Materials* **6**, 8795-8803 (2023). https://doi.org/10.1021/acsanm.3c01190

3 Ji, J., Yu, G., Xu, C. & Xiang, H. J. Fractional quantum ferroelectricity. *Nat. Commun.* **15**, 135 (2024). https://doi.org/10.1038/s41467-023-44453-y

4 Ding, W. *et al.* Prediction of intrinsic two-dimensional ferroelectrics in In_2Se_3 and other III_2-VI_3 van der Waals materials. *Nat. Commun.* **8**, 14956 (2017). https://doi.org/10.1038/ncomms14956

5 Liu, L. *et al.* Atomically resolving polymorphs and crystal structures of In_2Se_3. *Chem. Mater.* **31**, 10143-10149 (2019). https://doi.org/10.1021/acs.chemmater.9b03499

6 Yannopoulos, S. N. & Andrikopoulos, K. S. Raman scattering study on structural and dynamical features of noncrystalline selenium. *J. Chem. Phys.* **121**, 4747-4758 (2004). https://doi.org/10.1063/1.1780151

7 Sader, J. E. Frequency response of cantilever beams immersed in viscous fluids with applications to the atomic force microscope. *J. Appl. Phys.* **84**, 64-76 (1998). https://doi.org/10.1063/1.368002

8 Xue, F. *et al.* Multidirection piezoelectricity in mono- and multilayered hexagonal α-In_2Se_3. *ACS Nano* **12**, 4976-4983 (2018). https://doi.org/10.1021/acsnano.8b02152

9 Jiang, X. *et al.* Strong piezoelectricity and improved rectifier properties in mono- and multilayered $CuInP_2S_6$. *Adv. Funct. Mater.* **33**, 2213561 (2023). https://doi.org/10.1002/adfm.202213561

10 Hallil, H. *et al.* Strong piezoelectricity in 3R-MoS_2 flakes. *Adv. Electron. Mater.* **8**, 2101131 (2022). https://doi.org/10.1002/aelm.202101131

11 Sui, F. *et al.* Sliding ferroelectricity in van der Waals layered γ-InSe semiconductor. *Nat. Commun.* **14**, 36 (2023). https://doi.org/10.1038/s41467-022-35490-0

12 Li, X. Q. *et al.* Epitaxial strain enhanced ferroelectric polarization toward a giant tunneling electroresistance. *ACS Nano* **18**, 7989-8001 (2024). https://doi.org/10.1021/acsnano.3c10933

13 Chi, Y., Sun, Z.-D., Xu, Q.-T., Xue, H.-G. & Guo, S.-P. Hexagonal In_2Se_3: A defect Wurtzite-type infrared nonlinear optical material with moderate birefringence contributed by unique $InSe_5$ unit. *ACS Appl. Mater. Interfaces* **12**, 17699-17705 (2020). https://doi.org/10.1021/acsami.9b23085
